# Glucocorticoid affects dendritic transport of BDNF-containing vesicles

**DOI:** 10.1038/srep12684

**Published:** 2015-08-04

**Authors:** Naoki Adachi, Tadahiro Numakawa, Shingo Nakajima, Masashi Fukuoka, Haruki Odaka, Yusuke Katanuma, Yoshiko Ooshima, Hirohiko Hohjoh, Hiroshi Kunugi

**Affiliations:** 1 Department of Mental Disorder Research, National Institute of Neuroscience, National Center of Neurology and Psychiatry, Tokyo, Japan; 2 Core Research for Evolutional Science and Technology Program (CREST), Japan Science and Technology Agency (JST), Saitama, Japan; 3 Department of Biomedical Chemistry, Kwansei Gakuin University, Sanda, Japan; 4 Department of Cell Modulation, Institute of Molecular Embryology and Genetics, Kumamoto University, Kumamoto, Japan; 5 Faculty of Health Science, Hokkaido University, Sapporo, Japan; 6 Department of Molecular Pharmacology, National Institute of Neuroscience, National Center of Neurology and Psychiatry, Tokyo, Japan; 7 Department of Life Science and Medical Bioscience, School of Advanced Science and Engineering, Waseda University, Tokyo, Japan

## Abstract

Brain-derived neurotrophic factor (BDNF) is essential for neuronal survival, differentiation, and functions in the central nervous system (CNS). Because BDNF protein is sorted into secretory vesicles at the trans-Golgi network in the cell body after translation, transport of BDNF-containing vesicles to the secretion sites is an important process for its function. Here we examined the effect of dexamethasone (DEX), a synthetic glucocorticoid, on BDNF-containing vesicle transport and found that DEX decreased the proportion of stationary vesicles and increased velocity of the microtubule-based vesicle transport in dendrites of cortical neurons. Furthermore, DEX increased huntingtin (Htt) protein levels via glucocorticoid receptor (GR) activation, and reduction in the amount of Htt by a specific shRNA reversed the action of DEX on BDNF vesicle transport. Given that Htt protein is a positive regulator for the microtubule-dependent vesicular transport in neurons, our data suggest that glucocorticoid stimulates BDNF vesicle transport through upregulation of Htt protein levels.

Brain-derived neurotrophic factor (BDNF), a member of neurotrophin family, has several important roles in the central nervous system (CNS) of both developing and mature brain by supporting neuronal survival, facilitating neurite outgrowth, and regulating synaptic plasticity^1^,^2^,^3^,^4^. BDNF dominantly binds to a specific tropomyosin-related kinase receptor, TrkB, to stimulate mainly three intracellular signaling pathways; mitogen-activated protein kinase/extracellular signal-regulated protein kinase (MAPK/ERK), phosphatidylinositol 3-kinase (PI3K), and phospholipase Cγ (PLCγ) pathways^1^,^2^,^3^,^4^,^5^. Both autocrine and paracrine actions of BDNF have been evident through a neuronal activity-dependent exocytic fusion of BDNF-containing large dense core vesicles (LDCV) from dendrites and axons^6^,^7^,^8^.

BDNF protein can be synthesized within the cell body and locally at dendrites and transported distally and proximally in neurites, which has been revealed by expressing fluorescent protein-tagged BDNF in cultured neurons^6^,^9^,^10^,^11^,^12^. The intracellular transport of BDNF-containing vesicles is carried out by kinesin and dynein motor protein complex moving on microtubules (MTs) distributed throughout the neuron^13^,^14^. Recent studies have revealed that huntingtin (Htt) and huntingtin-associated protein (HAP) also constitute the motor protein complex to facilitate vesicular transport bi-directionally in axons^13^,^15^ and dendrites^16^. Htt regulates BDNF mRNA transcription^17^ and supports the BDNF-containing vesicle transport^13^,^18^,^19^. Its mutation producing abnormal Htt proteins with polyglutamine expansion (polyQ) is causal for Huntington’s disease (HD), a neurodegenerative disease in the striatal neurons that presents progressive psychiatric, cognitive and motor dysfunction. The impairment of the trophic support of BDNF from cortical neurons caused by loss of function of wild-type Htt is thought to be one of the main factors of neuronal cell death in the striatum of HD patients^13^,^18^,^20^.

Glucocorticoids are steroid hormones secreted from the adrenal cortex in response to stressful stimuli and have endocrinological coping functions to protect the normal defense reactions that are stimulated by stress from its excessive reaction^21^. Secretion of glucocorticoids is regulated by the hypothalamic-pituitary-adrenal (HPA) axis that consists of a sequential secretion/stimulation of hormones; corticotropin-releasing hormone (CRH) from the paraventricular nucleus (PVN), CRH-trigged adrenocorticotropic hormone (ACTH) from the anterior pituitary, and ACTH-induced glucocorticoids from the adrenal glands^5^,^22^,^23^. Previous studies have reported that elevated circulating glucocorticoid levels due to impaired negative feedback of the HPA axis is observed in patients with major depression^24^,^25^,^26^, and impaired BDNF function may be involved in the pathophysiology of major depression^5^,^27^,^28^,^29^. Furthermore, glucocorticoids induced by stressful event have effects on learning and memory^30^ as well as BDNF. Although recent studies have revealed that glucocorticoid regulates the process of transcription and secretion of BDNF^31^, little is known whether intracellular transport of BDNF vesicles is affected by glucocorticoids. In this study, we investigated whether a synthetic glucocorticoid dexamethasone (DEX), a specific agonist for glucocorticoid receptor (GR), affects intracellular BDNF transport in neurites. We found that DEX treatment facilitated trafficking of BDNF-containing vesicles in dendrites through increasing Htt protein expression levels in cultured cortical neurons.

## Results

### Microtubule-dependent transport of BDNF-GFP-containing vesicles in cortical neurons

**Figure 1:**
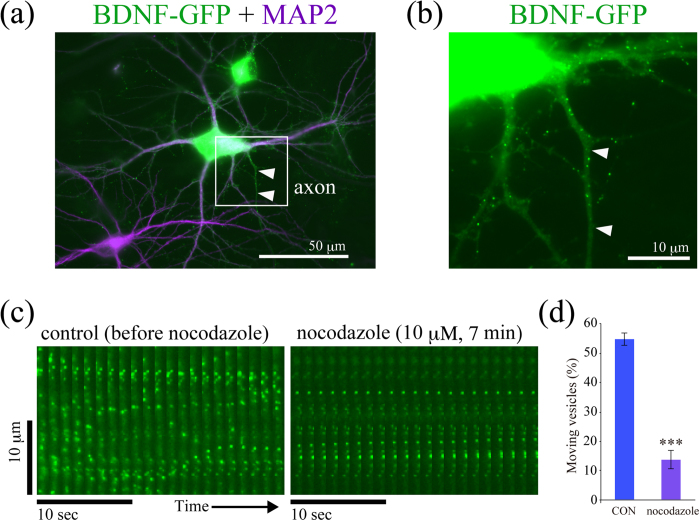
A vesicular expression of BDNF-GFP in cultured cortical neurons and microtubule-dependent trafficking of BDNF-GFP-containing vesicles. (**a**) A representative image of BDNF-GFP-expressing cortical neuron (green, DIV 12). Axon is distinguishable from dendrites by immunostaining with MAP2 (purple) which specifically expresses in the cell body and dendrites of neurons. Arrowheads indicate axon (MAP2-negative). (**b**) A magnified image of the white square region of (**a**) without MAP2 image. (**c**) Nocodazole (10 μM), a microtubule polymerization inhibitor, blocked trafficking of BDNF-GFP vesicles in dendrite. Two sets of twenty-one sequential time-lapse images obtained from a part of a dendrite before and after nocodazole treatment are arranged in a horizontal direction. (**d**) Quantitative analysis of the proportion of moving BDNF vesicles before and after nocodazole treatment. The proportion at 7 minutes after nocodazole application was dramatically reduced compared to that at 1 minute before the treatment. Data were obtained from 5 neurons from two independent culture preparations including 170 and 142 vesicles before and after nocodazole treatment, respectively. ***P < 0.001 vs. CON (before treatment). Statistical significance was evaluated by student’s *t*-test.

Cultured cortical neurons were transfected with plasmids encoding BDNF tagged with GFP at DIV 10–11. A vesicular expression of BDNF-GFP in neurons was observed 16 hours after transfection as described previously^9^, and time-lapse imaging and immunostaining experiments were conducted 18 hours after transfection (Supplementary video 1, 2). A similar distribution pattern of GFP-tagged BDNF to that of endogenous BDNF was previously confirmed^9^. We considered one neurite where MAP2 immunoreactivity is vanishingly low as axon and the others as dendrites (Fig. 1a,b). To support this, a cortical neuron had one neurite where Tau-1 (known for its distribution in axons) substantially expressed and MAP2 immunoreactivity is very low (Supplementary Figure S1). To determine whether trafficking of BDNF-containing vesicles in dendrites is dependent on MTs, nocodazole (an inhibitor of MTs polymerization) was applied to neurons expressing BDNF-GFP (Fig. 1c). Trafficking of BDNF-GFP vesicles was disrupted by 10 μM nocodazole exposure within 10 min (Fig. 1c,d), suggesting that BDNF-GFP vesicle transport in dendrites was microtubule-dependent.

### Dexamethasone stimulated BDNF vesicle transport in dendrites via GR activation

**Figure 2:**
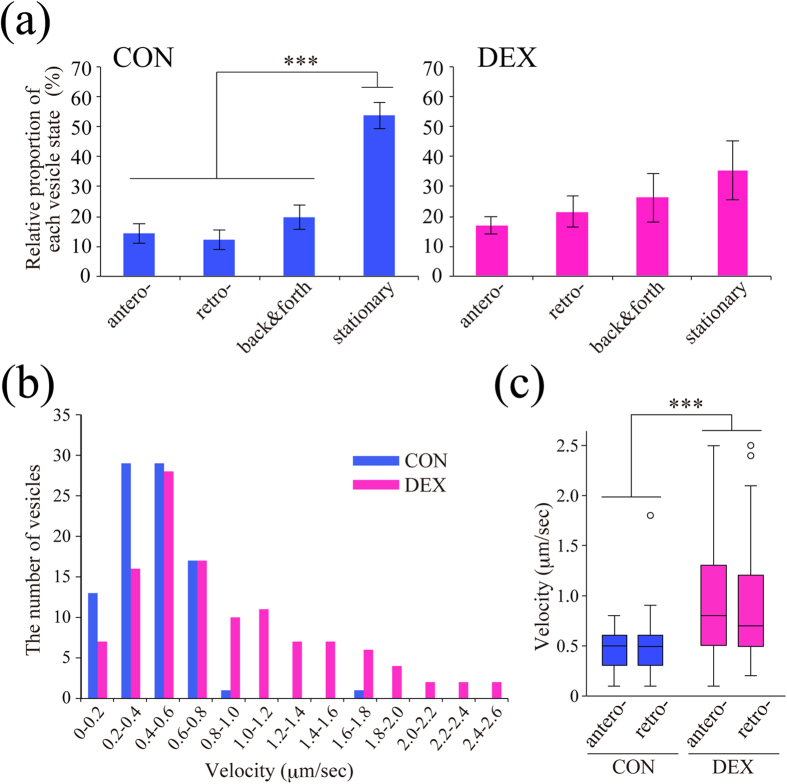
DEX stimulated the dendritic transport of BDNF vesicles in cortical neurons. (**a**) About 50% of BDNF-containing vesicles were stationary (not moving) in dendrites of cortical neurons (DIV 11–12) while DEX (1 μM, 24 h) stimulated transport of the vesicles. Data represent mean ± SEM. Data were obtained from 7 (CON) and 6 (DEX) neurons from four independent culture preparations including 525 and 423 vesicles, respectively. ***P < 0.001 for the difference between moving (antero-, retro-grade, and back & forth) vs. stationary vesicles. Statistical significance was evaluated by student’s *t*-test. (**b**) Non-normal distribution of velocities of BDNF-containing vesicles both in control and DEX-treated conditions. Velocities were obtained from 90 (CON) and 119 (DEX) vesicles of 7 (CON) and 6 (DEX) neurons from four independent culture preparations. Each velocity range represents the range of greater than the lower value to less than or equal to the higher value. (c) DEX increased velocity of BDNF vesicle transport in both anterograde and retrograde directions. CON anterograde (median 0.5 μm/sec, percentiles 0.3–0.6); CON retrograde (median 0.5 μm/sec, percentiles 0.3–0.6); DEX anterograde (median 0.8 μm/sec, percentiles 0.5–1.3); DEX retrograde (median 0.7 μm/sec, percentiles 0.5–1.25). Circles outside the whiskers indicate outliers. ***P < 0.001 vs. CON (without DEX), Kruskal-Wallis test.

**Figure 3:**
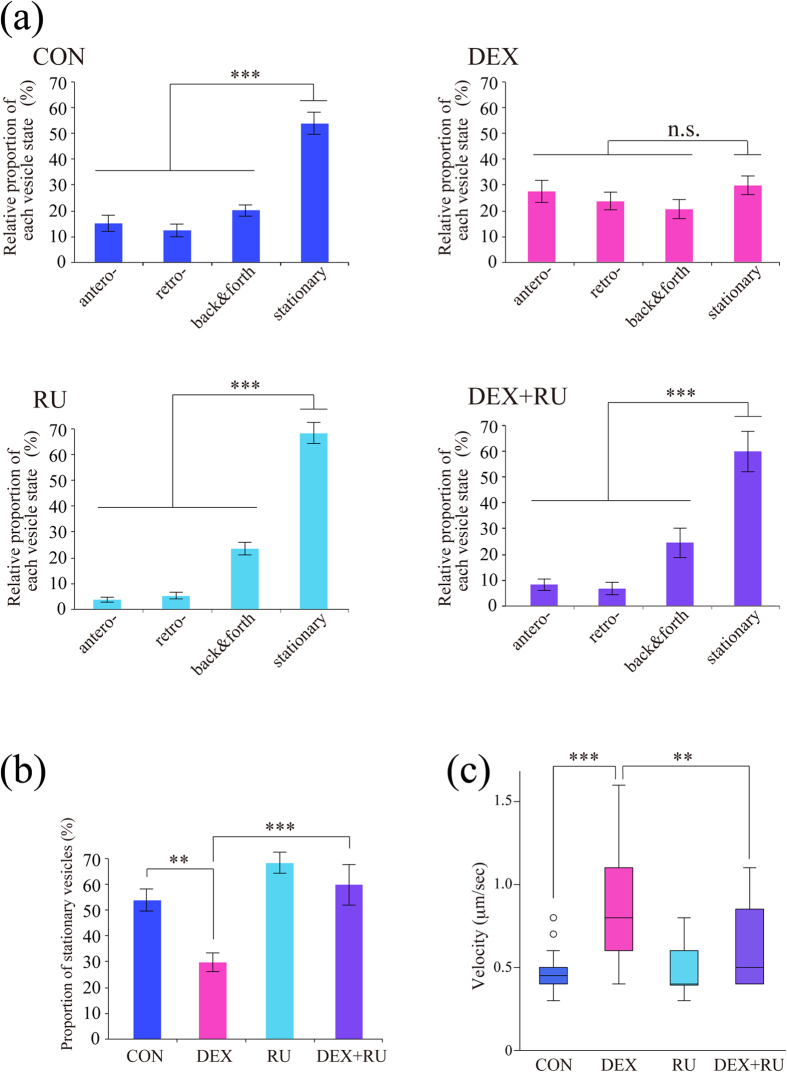
DEX changed transport properties of BDNF vesicle via GR function. (**a**) DEX-induced enhancement of BDNF vesicle transport was blocked by RU486 (mifepristone, a GR antagonist). Data were obtained from 8 (CON), 6 (DEX), 7 (RU), and 6 (DEX + RU) neurons from three independent culture preparations including 302, 181, 258, and 196 vesicles, respectively. ***P < 0.001 for the difference between moving (antero-, retro-grade, and back & forth) vs. stationary vesicles, student’s *t*-test. (**b**) The proportion of stationary vesicles in each condition was summarized. ***P < 0.001, **P < 0.01. Statistical significance was evaluated by two-way ANOVA followed by Bonferroni *post-hoc* test. (**c**) RU486 partially suppressed the increased velocity of BDNF vesicle transport after DEX treatment. Velocities were obtained from 8 (CON), 6 (DEX), 7 (RU), and 6 (DEX + RU) neurons from three independent culture preparations including 40 (CON), 37 (DEX), 23 (RU), and 20 (DEX + RU) vesicles. CON (median 0.45 μm/sec, percentiles 0.4–0.5); DEX (median 0.8 μm/sec, percentiles 0.6–1.1); RU (median 0.4 μm/sec, percentiles 0.4–0.6); DEX + RU (median 0.5 μm/sec, percentiles 0.4–0.88). Circles outside the whiskers indicate outliers. ***P < 0.001, **P < 0.01, Kruskal-Wallis test.

We then examined effect of DEX, a synthetic GR agonist, on BDNF vesicle trafficking. As previously reported^9^, about 50% of vesicles were stationary (not moving), 20% showed back and forth movement within 10 μm, and the remaining vesicles did antero- or retrograde movement in dendrites at DIV 11–12 (Fig. 2a, Supplementary video 1, 2). Such BDNF vesicle transport properties in cortical neurons were changed by 1 μM DEX treatment for 24 hours to be more dynamic (Fig. 2a, Supplementary video 3). DEX decreased the relative proportion of the stationary vesicles and increased that of moving and trafficking vesicles (Fig. 2a). Interestingly, velocities of BDNF vesicle transport in both antero- and retrograde directions were significantly increased after DEX treatment although it was not normally distributed in both control and DEX-treated conditions: CON anterograde (median 0.5 μm/sec, percentiles 0.3–0.6); CON retrograde (median 0.5 μm/sec, percentiles 0.3–0.6); DEX anterograde (median 0.8 μm/sec, percentiles 0.5–1.3); DEX retrograde (median 0.7 μm/sec, percentiles 0.5–1.25) (Fig. 2b,c). The DEX-induced transport-enhancing effect was canceled when 1 μM RU486 (mifepristone, a GR antagonist) was applied to cultured neurons 20 min prior to DEX administration (Fig. 3a,b). The increased velocity of BDNF vesicle transport by DEX was also partially suppressed by RU486: CON (median 0.45 μm/sec, percentiles 0.4–0.5); DEX (median 0.8 μm/sec, percentiles 0.6–1.1); RU (median 0.4 μm/sec, percentiles 0.4–0.6); DEX + RU (median 0.5 μm/sec, percentiles 0.4–0.88) (Fig. 3c), suggesting that GR activation was involved in the enhancement of BDNF vesicle transport by DEX.

### DEX did not affect the localization of BDNF vesicles at postsynaptic sites

To evaluate whether DEX affects the number of synaptic sites and co-localization of the stationary vesicles with presynaptic sites, cultured neurons expressing BDNF-GFP were immunostained with an antibody for synaptotagmin, a presynaptic marker, after 48 hours of DEX treatment (Supplementary Figure S2a) because we speculated that the stationary vesicles might be localized at postsynaptic sites. Neither DEX nor RU486 changed the number of presynaptic sites (Supplementary Figure S2b). Even in control neurons, only 7% of the stationary BDNF-containing vesicles localized in the vicinity of the postsynaptic sites faced to synaptotagmin-positive presynapses (Supplementary Figure S2c), and the co-localization rate was not significantly changed by DEX or RU486 treatment (Supplementary Figure S2c).

### DEX-increased Htt protein levels contributed to the effect of DEX on BDNF vesicle transport

**Figure 4:**
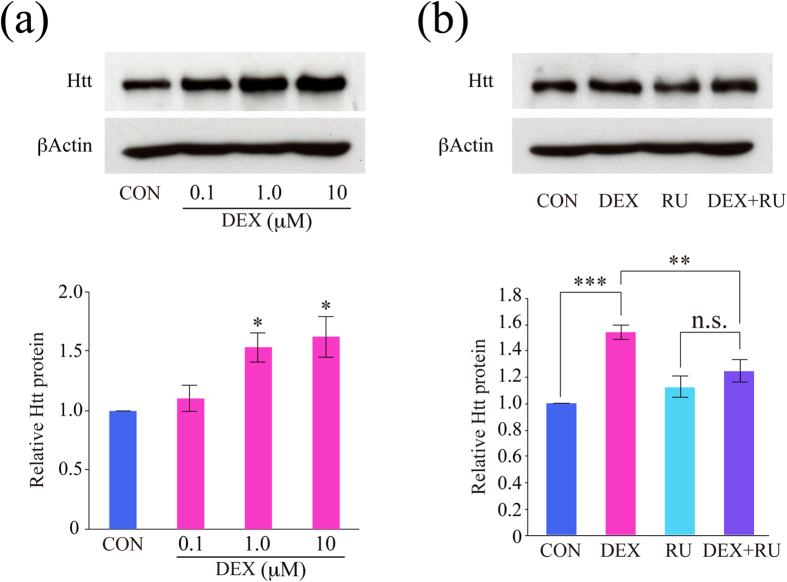
DEX increased Htt protein levels through GR. (**a**) DEX (1 and 10 μM, for 24 h) significantly increased Htt expression in cortical neurons. (n = 5). *P < 0.05 vs. CON, one-way ANOVA followed by Bonferroni *post-hoc* test. (**b**) Pretreatment with RU486 blocked the effect of DEX on Htt expression levels (n = 8). ***P < 0.001, **P < 0.01, n.s. means not significantly different (*P* > 0.05), two-way ANOVA followed by Bonferroni *post-hoc* test. Htt: huntingtin. The gels have been run under the same experimental condition in each experiment. Original blot images before cropping are shown in supplementary figure S3.

**Figure 5:**
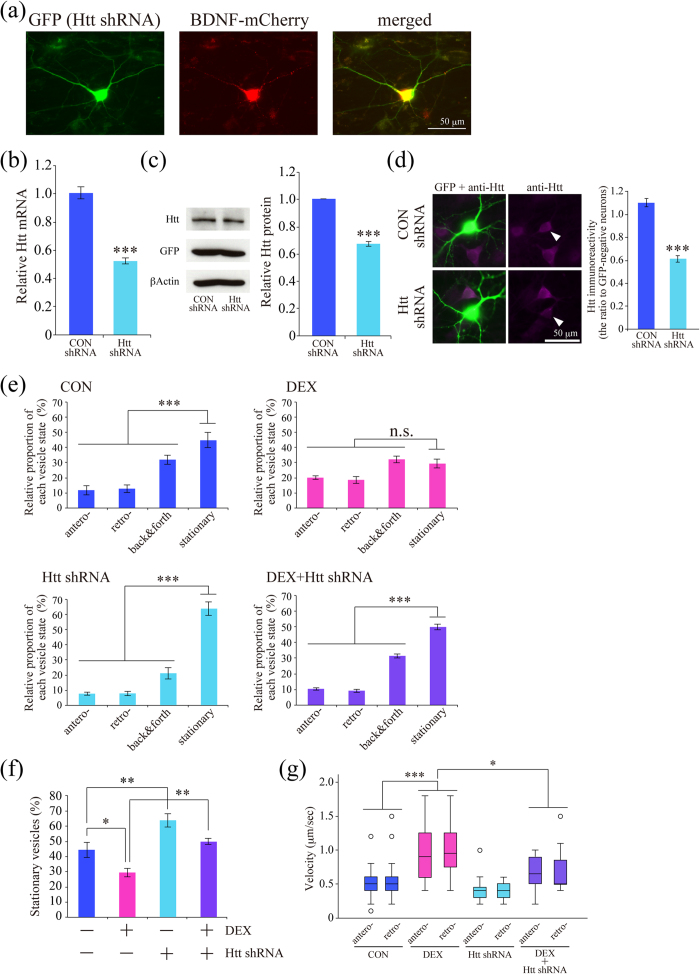
Downregulation of Htt inhibited the effect of DEX on BDNF vesicle transport. (**a**) A representative cortical neuron expressing both Htt shRNA with GFP and BDNF-mCherry. (**b,c**) The function of Htt shRNA was confirmed with PC12D cells about suppression of Htt mRNA levels (n = 5) (**b**) and protein levels (n = 5) (**c**) at 48 hours after transfection. ***P < 0.001 vs. CON, student’s *t*-test. The gels have been run under the same experimental condition. Original blot images before cropping are shown in supplementary figure S3. (**d**) Immunoreactivity of Htt was reduced in Htt shRNA expressing neurons. Cortical neurons were stained with anti-Htt antibody (purple). Relative Htt immunoreactivity in the cell body of Htt shRNA expressing neurons was obtained as the ratio between GFP-positive neuron and adjacent GFP-negative neurons. Data from 33 (CON shRNA) and 31 (Htt shRNA) pairs of neurons from three independent culture preparations were shown. ***P < 0.001 vs. control shRNA, student’s *t*-test. (**e,f,g**) Htt shRNA transfection reversed the DEX effect on BDNF vesicle transport in basic transport properties (**e,f**) and velocity (**g**). Data were obtained from 6 (CON), 6 (DEX), 6 (Htt shRNA), and 6 (DEX + Htt shRNA) neurons from two independent culture preparations including 220, 204, 168, and 257 vesicles, respectively. (**e**) ***P < 0.001 for the difference between moving (antero-, retro-grade, and back & forth) vs. stationary vesicles, student’s *t*-test. (**f**) The proportion of stationary vesicles in each condition was summarized. **P < 0.01, *P < 0.05 two-way ANOVA followed by Bonferroni *post-hoc* test. (**g**) Velocities were obtained from 47 (CON), 51 (DEX), 33 (Htt shRNA), and 49 (DEX + Htt shRNA) vesicles 6 (CON), 6 (DEX), 6 (Htt shRNA), and 6 (DEX + Htt shRNA) neurons from two independent culture preparations. CON anterograde (median 0.5 μm/sec, percentiles 0.4–0.6); CON retrograde (median 0.5 μm/sec, percentiles 0.4–0.6); DEX anterograde (median 0.9 μm/sec, percentiles 0.6–1.3); DEX retrograde (median 0.95 μm/sec, percentiles 0.73–1.3); Htt shRNA anterograde (median 0.4 μm/sec, percentiles 0.3–0.48); Htt shRNA retrograde (median 0.4 μm/sec, percentiles 0.3–0.5); DEX + Htt shRNA anterograde (median 0.65 μm/sec, percentiles 0.5–0.9); DEX + Htt shRNA retrograde (median 0.5 μm/sec, percentiles 0.5–0.9). Circles outside the whiskers indicate outliers. ***P < 0.001, *P < 0.05, Kruskal-Wallis test.

Next we determined expression levels of Htt protein, and found that Htt level was increased by 24-hour DEX treatment in a dose-dependent manner (Fig. 4a). DEX (1 μM)-induced Htt upregulation was inhibited by 1 μM RU486 (Fig. 4b), indicating that DEX increased Htt protein levels through GR function. To confirm that DEX-induced enhancement of BDNF vesicle transport is dependent on the upregulation of Htt expression, plasmid encoding Htt shRNA and GFP was co-transfected with mCherry-tagged BDNF (Fig. 5a). Reduced Htt mRNA and protein levels by approximately 40% were observed 24 hours after Htt shRNA transfection both in PC12D cells (Fig. 5b,c) and cortical neurons (Fig. 5d). When Htt shRNA was transfected into cortical neurons, DEX-dependent enhancement of BDNF vesicle transport was reversed, similar to the properties observed in control neurons (Fig. 5e). DEX-induced reduction in the relative proportion of stationary vesicles was returned to the control levels by Htt shRNA (Fig. 5f) Importantly, Htt shRNA transfection without DEX significantly increased the proportion of stationary vesicles (Fig. 5f). The increased velocity of transport by DEX treatment was at least partially slowed by the Htt downregulation in both antero- and retrograde directions: CON anterograde (median 0.5 μm/sec, percentiles 0.4–0.6); CON retrograde (median 0.5 μm/sec, percentiles 0.4–0.6); DEX anterograde (median 0.9 μm/sec, percentiles 0.6–1.3); DEX retrograde (median 0.95 μm/sec, percentiles 0.73–1.3); Htt shRNA anterograde (median 0.4 μm/sec, percentiles 0.3–0.48); Htt shRNA retrograde (median 0.4 μm/sec, percentiles 0.3–0.5); DEX + Htt shRNA anterograde (median 0.65 μm/sec, percentiles 0.5–0.9); DEX + Htt shRNA retrograde (median 0.5 μm/sec, percentiles 0.5–0.9) (Fig. 5g).

## Discussion

Recent studies have unveiled beneficial biological roles of wild-type Htt protein as a regulator for transcription and vesicular transport in the CNS while mutation (poly Q expansion) in exon I of the human Htt gene had been presumed to cause aggregation of mutant Htt proteins and result in a toxic gain of function in striatal neurons^32^. Zuccato *et al.* firstly reported that wild type Htt protein positively regulated BDNF transcription in cortical neurons whereas mutant Htt suppressed^17^. Another Htt function in vesicular transport was subsequently revealed. Htt associates with the microtubule-based motor complex including htt-associated protein-1 (HAP1) and kinesin 1 for anterograde and HAP1, p150Glued, dynactin and dynein for retrograde transport^33^,^34^,^35^ to support trafficking of vesicles containing proteins^13^ and mRNA^16^. Gauthier *et al.* showed that overexpression of wild-type Htt increased velocity of BDNF vesicle transport and decreased the proportion of static (stationary) vesicles while siRNA knockdown of Htt caused the opposite in neuroblastoma cells and cultured cortical neurons^13^. Ma *et al.* recently showed that specific shRNA-induced knockdown of Htt impaired transport of β-actin mRNA-containing ribonucleoprotein particles (RNPs) into dendrites and caused accumulation of the mRNA within the cell body^16^. In the present study, we showed a novel regulation of glucocorticoid in BDNF vesicle transport. Consistent with previous studies^13^,^19^, BDNF-containing vesicle trafficking was diminished by nocodazole, suggesting that BDNF vesicle transport in dendrites of cortical neurons is microtubule-based. DEX increased Htt protein expression levels in cultured cortical neurons and promoted BDNF vesicle transport. GR activation was required to induce upregulation of Htt and stimulation of vesicular transport. GR regulates transcription of genes containing the glucocorticoid response element (GRE) in their promoter region^36^,^37^. However, the GRE sequence has not been identified in the regulatory region of the Htt gene, suggesting that GR may indirectly increase the amount of intracellular Htt protein. Furthermore, RU486 partially inhibited the effect of DEX on the velocity of transport whereas the proportion of stationary vesicles was almost completely reversed. Considering that RU486 suppressed DEX-induced upregulation of Htt completely, DEX could also accelerate the velocity via a Htt-independent mechanism. This speculation is also supported by the result that shRNA-induced decline in Htt protein did not completely reduce the velocity increased by DEX.

There are various phosphorylation sites identified in Htt protein^38^. Phosphorylation at serine 421 (S421) facilitates to recruit kinesin-1 to vesicles and MTs, which in turn moves BDNF-containing vesicles anterogradely^19^. Reduction of S421 phosphorylation, on the contrary, stimulates retrograde transport^19^. Because DEX affected BDNF vesicle transport similarly in both directions, S421 phosphorylation of Htt would not be involved in the DEX effect.

Kino and colleagues reported interesting glucocorticoid regulation on the transcription and secretion of BDNF^39^. DEX reduced intracellular BDNF mRNA levels and the amount of secreted BDNF protein in cultured cortical neurons^39^. Because a putative GRE site is identified in the promoter region of BDNF exon VI, a direct regulation by GR has been suggested. Phosphorylation of GR by cyclin-dependent kinase 5 (CDK5) enhanced the DEX effect on BDNF transcription^39^. DEX-induced enhancement in the BDNF-containing vesicle transport reported in the present study, therefore, might act as a compensational supplementation of BDNF to the secretion sites after glucocorticoid exposure.

Dendritic localization of BDNF mRNA is also regulated by 5′ splice variants^40^ or 3′ untranslated regions (3′ UTRs)^41^ of the mRNA in hippocampal neurons. Furthermore, a part of dendritic translocation of BDNF mRNA is enhanced by neuronal activity through translin-dependent mechanisms^42^. Although it is still not clear whether BDNF mRNAs are packaged into secretory granules, transport of BDNF mRNAs might also be affected by Htt and glucocorticoids if the mRNAs are transported by the RNPs like β-actin mRNA^16^. Co-localization of BDNF mRNA with Htt protein^43^ may support the speculation.

Against our expectations, the majority of stationary BDNF vesicles were not located around postsynaptic sites. DEX increased the number of static vesicles located at extrasynaptic regions to move while it did not affect the proportion of stationary vesicles in the vicinity of postsynaptic sites. Further studies are needed to clarify whether stationary vesicles are equipped at BDNF releasing sites in neurites and what kind of functions are fulfilled by back-and-forth vesicles. We recently reported that TrkB and GR make a complex that contributes to recruit PLCγ to TrkB in immature cortical neurons^44^. The number of GR was declined by binding of glucocorticoids probably through degradation in the nucleus after playing its role as a transcription factor, which in turn resulted in attenuated BDNF-induced PLCγ activation^44^. Furthermore, DEX inhibited BDNF-dependent synaptic maturation in developing hippocampal neurons by suppressing BDNF-induced ERK activation^45^. Considering these adverse effects of glucocorticoids on BDNF/TrkB signaling pathways in immature neurons, the promoting effect of DEX on BDNF vesicle transport in this study seems to be inconsistent. DEX-stimulated BDNF vesicle transport in maturated neurons, however, might occur to compensate reduced BDNF/TrkB function or the inconsistency might reflect diverse glucocorticoids’ effects depending on developmental stage of neurons. DEX has also been shown to induce TrkB phosphorylation in the rat hippocampus and cultured hippocampal neurons^46^. Taken together, reciprocal action of BDNF/TrkB and glucocorticoids is increasingly of interest^5^,^31^ and detail studies with different regions of the brain and developmental stages would be required to reveal more precise functional interaction between the endocrine and central nervous systems.

## Methods

### Cell culture and drug treatment

Primary cortical neurons were prepared from cortical tissues of postnatal 1- or 2-day-old Wistar rats (SLC, Shizuoka, Japan) as described previously^47^. Briefly, dissociated cells were cultured on polyethyleneimine-coated culture dishes (BD Falcon, NJ) at a 5 × 10^5^/cm^2^ density with the 5/5 DF culture medium consisting of 5% fetal bovine serum (FBS), 5% heated-inactivated horse serum (HS), 90% of a 1:1 mixture of Dulbecco’s modified Eagle’s medium (DMEM) and Ham’s F-12 medium (Gibco, Life Technologies, NY,) with 50 U/ml penicillin and streptomycin (Gibco), for immunoblotting and quantitative PCR. For real-time imaging and immunocytochemistry, cortical neurons were plated onto a previously prepared astroglial feeder layer formed on polylysine-coated glass bottom dishes (Matsunami, Osaka, Japan), as described previously^9^. PC12D cells were grown in high glucose DMEM (Gibco) containing 10% FBS, 5% HS and 50 U/ml penicillin/streptomycin according to^48^. Cultured neurons were treated with DEX (1 μM, Sigma, MO) at 11–12 days *in vitro* (DIV) for 24 hours. RU486 (Mifepristone, 1 μM, LKT Laboratories Inc., MN) was applied 20 min before starting DEX treatment. DEX and RU486 were dissolved in dimethyl sulfoxide (DMSO), and the same concentration of DMSO was applied as vehicle control for each experiment.

### Immunoblotting

Cultured neurons were lysed with SDS lysis buffer containing 1% SDS, 20 mM Tris-HCl (pH 7.4), 5 mM EDTA (pH 8.0), 10 mM NaF, 2 mM Na_3_VO_4_, 0.5 mM phenylarsine oxide, and 1 mM phenylmethylsulfonyl fluoride. After quantification of protein concentration, equal amounts of the total proteins were separated by SDS-polyacrylamide gels (8% acrylamide) and transferred to polyvinylidene fluoride (PVDF) membranes (Merck Millipore, MA). The membranes were blocked with StartingBlock Blocking Buffers (Thermo Scientific, NH) for 1 h at room temperature, followed by incubation with antibodies diluted in 1% fat-free powdered milk (Megmilk Snow Brand Co Ltd, Saitama, Japan). Primary antibodies were used at the following dilutions: anti-Huntingtin (clone 1HU-4C8) (1:1000, Merck Millipore), anti-GFP (1:1000, MBL, MA), and anti-βactin (1:5000, Sigma). The immunoreactivity of bands was quantified using Lane & Spot Analyzer software (ATTO Corporation, Tokyo, Japan).

### DNA constructs and transfection

The pEGFP-N1 vector (Clontech, CA)-based plasmid expressing prepro-mouse BDNF tagged with GFP at the C-terminus was provided by Dr. Masami Kojima^49^. The BDNF-mcherry construct was made by subcloning of the prepro-mouse BDNF cDNA into the multiple cloning site of the pmCherry-N1 vector (Clontech) at EcoRI-BamHI site. For Htt knockdown, Oligonucleotide DNA sequences encoding shRNAs against *Htt* were designed, chemically synthesized and inserted into pcDNA™ 6.2-GW/EmGFP-miR expression vector using a BLOCK-iT^TM^ Pol II miR RNAi Expression Vector kit with EmGFP (Life Technologies) according to the manufacturer’s instructions. The pcDNA™ 6.2-GW/EmGFP-miR-neg control plasmid provided by the manufacture was used as a negative control. The sequences of the designed DNAs were as follows:

BL-Rat.Htt-Ss:

TGCTGTTCATCAGTTTTTCCAGGGTTGTTTTGGCCACTGACTGACAACCCTGGAAACTGATGAT

BL-Rat.Htt-As:CCTGATCATCAGTTTCCAGGGTTGTCAGTCAGTGGCCAAAACAACCCTGGAAAAACTGATGAAC

Cortical neurons or PC12D cells were transfected with mixture of each plasmid DNA (1 μg) and Lipofectamine 2000 (3 μl) (Invitrogen, CA) in OptiMEM (Invitrogen) per dish, according to the manufacturer’s protocol.

### Live-cell imaging

Images of neurons expressing fluorescent protein-tagged BDNF were captured using a Axio Observer Z1 fluorescence microscope system equipped with a Plan-Apochomat 40x/1.3 oil immersion objective and a Colibri LED light source (Carl Zeiss, Westlar, Germany). Time-lapse recordings were carried out 18–24 h after transfection of fluorescent protein-tagged BDNF plasmid. During time-lapse imaging, cultured neurons were perfused with the buffer containing (in mM): 120 NaCl, 1.2 KH_2_PO_4_, 2 CaCl_2_, 1 MgSO_4_, 30 glucose, and 20 N-2-hydroxyethylpiperazine-N’-2-ethanesulfonic acid (HEPES; pH 7.4), which was incubated at 37 ^o^C. Sequential images were acquired using a cooled CCD camera (Carl Zeiss) with about 1 s interval and 200–600 ms exposure time. The movement of BDNF-fluorescent protein positive vesicles was analyzed by tracking the positions of EGFP or mCherry in dendrites as a function of time using the Axiovision software (Carl Zeiss).

### Immunocytochemistry

Cortical neurons cultured in the glass bottom dish were fixed with Dulbecco’s phosphate-buffered saline (PBS)-based 4% paraformaldehyde (Sigma) solution containing 4% sucrose for 20 min at room temperature. The cells were treated with 0.2% Triton-X (Sigma)/PBS for 5 min and blocked by 10% goat serum in PBS for 1 h at 37 ^o^C. Cells were then incubated with anti-MAP2 monoclonal antibody (isotype: IgG1, 1:250, Sigma), anti-Tau-1 monoclonal antibody (isotype: IgG2a, 1:500, Millipore), anti-Huntingtin antibody (clone HU-4E6) (1:1000, Merck Millipore), anti-synaptotagmin antibody (isotype: IgG2a, 1:250, Merck Millipore) overnight at 4 ^o^C. Secondary antibodies used in this study were Alexa Fluor 546 (1:2000, Molecular Probes, CA) and Alexa Fluor 647 (1:200, Molecular Probes). Fluorescent images were obtained using a Zeiss Observer Z1 microscope (Carl Zeiss, Oberkochen, Germany).

### Total RNA extraction, reverse transcription, and quantitative PCR

Total RNA of cultured cells was extracted using the mirvana miRNA Isolation Kit (Life Technologies) according to the manufacturer’s manual. cDNA of the sample was synthesized by reverse transcription with SuperScript VILO cDNA Synthesis kit (Invitrogen, CA). In real-time PCR analysis, the cDNA was amplified with specific TaqMan Gene Expression Assays (Rn00577462_m1 for rat Htt) (Applied Biosystems, MA) by ABI prism 7000 Sequence Detection System (Applied Biosystems). The Ct value of Htt mRNA was obtained using Sequence Detection System software (Applied Biosystems) and normalized with rat GAPDH control (4352338E) (Applied Biosystems).

### Statistical analysis

Quantified data are presented as mean ± standard error (SEM). Statistical significance was calculated using a one-way ANOVA followed by Bonferroni *post-hoc* test in SPSS ver.22 (SPSS Japan, Tokyo, Japan). The normality of the velocity data is checked by Kolmogorov-Smirnov test. For velocity data that are not normally distributed, non-parametric analysis Kruskal-Wallis test was conducted. In the box plots for not normally distributed data, the bottom boundary of the box indicates the 25th percentile, the line within the box represents the median, and the top boundary indicates the 75th percentile. The whiskers extend to 1.5 times the interquartile range or to the maximum or minimum values. Circles outside the whiskers indicate outliers. A probability value of less than 5% was considered statistically significant.

All methods were carried out in accordance with the approved guidelines of the National Institute of Neuroscience, National Center of Neurology and Psychiatry, Japan. All procedures were approved by the Animal Care and Use Committee of the National Institute of Neuroscience, National Center of Neurology and Psychiatry, Japan.

## Additional Information

**How to cite this article**: Adachi, N. *et al.* Glucocorticoid affects dendritic transport of BDNF-containing vesicles. *Sci. Rep.* **5**, 12684; doi: 10.1038/srep12684 (2015).

## Supplementary Material

Supplementary Information

Supplementary video 1

Supplementary video 2

Supplementary video 3
