# Supplementary material for: Glucocorticoid affects dendritic transport of BDNF-containing vesicles
Source: Sci Rep. 2015 Aug 4;5:12684. doi: 10.1038/srep12684 (PMC4523857; doi:10.1038/srep12684)

Supplementary Information

Title: Glucocorticoid affects dendritic transport of BDNF-containing vesicles

Authors: Naoki Adachi, Tadahiro Numakawa*, Shingo Nakajima, Masashi Fukuoka, Haruki Odaka, Yusuke Katanuma, Yoshiko Ooshima, Hirohiko Hohjoh, and Hiroshi Kunugi

Captions for Supplementary materials

Supplementary Figure S1. A representative images of a BDNF-GFP-expressing cortical neuron (green, DIV 13) immunostained with anti-MAP2 and Tau-1 antibodies. Note that one neurite where MAP2 immunoreactivity is vanishingly low (arrow heads) is Tau-1 positive, and the axonal process also contains BDNF-GFP vesicles.

Supplementary Figure S2. DEX did not affect the number of presynaptic sites and localization of BDNF vesicle at postsynaptic sites. (a) Cortical neurons expressing BDNF-GFP were immunostained with antibody for synaptotagmin as a presynaptic marker. (b) The number of synaptotagmin-positive presynaptic sites contacting at dendrites of BDNF-GFP-expressing neurons. Data represent mean ± SEM**.** (c) Percentage of BDNF-GFP-containing vesicles in the vicinity of the synaptotagmin-positive presynaptic sites. Data were obtained from 8 (CON), 6 (DEX), 7 (RU), and 6 (DEX+RU) neurons from three independent culture preparations including 302, 181, 258, and 196 vesicles, respectively.

Supplementary Figure S3. Western blot images before cropped to show in main figures. The areas cropped and shown in Figure 4 and Figure 5 are indicated by black squares.

Supplementary video 1. Intracellular transport of BDNF-GFP-containing vesicles in a rat cortical neuron was imaged for 2 minutes.

Supplementary video 2. Imaging of dendritic trafficking of BDNF-GFP-containing vesicles in control condition (same neuron as Supplementary video 1) is enlarged. Image width: 24 μm, 105 seconds imaging time.

Supplementary video 3. Imaging of dendritic trafficking of BDNF-GFP-containing vesicles in DEX-treated condition (1 μM, 24 hours). Image width: 24 μm, 105 seconds imaging time.

Title: Glucocorticoid affects dendritic transport of BDNF-containing vesicles

Naoki Adachi, Tadahiro Numakawa, Shingo Nakajima, Masashi Fukuoka, Haruki Odaka,
Yusuke Katanuma, Yoshiko Ooshima, Hirohiko Hohjoh, and Hiroshi Kunugi

Supplementary Figure S1

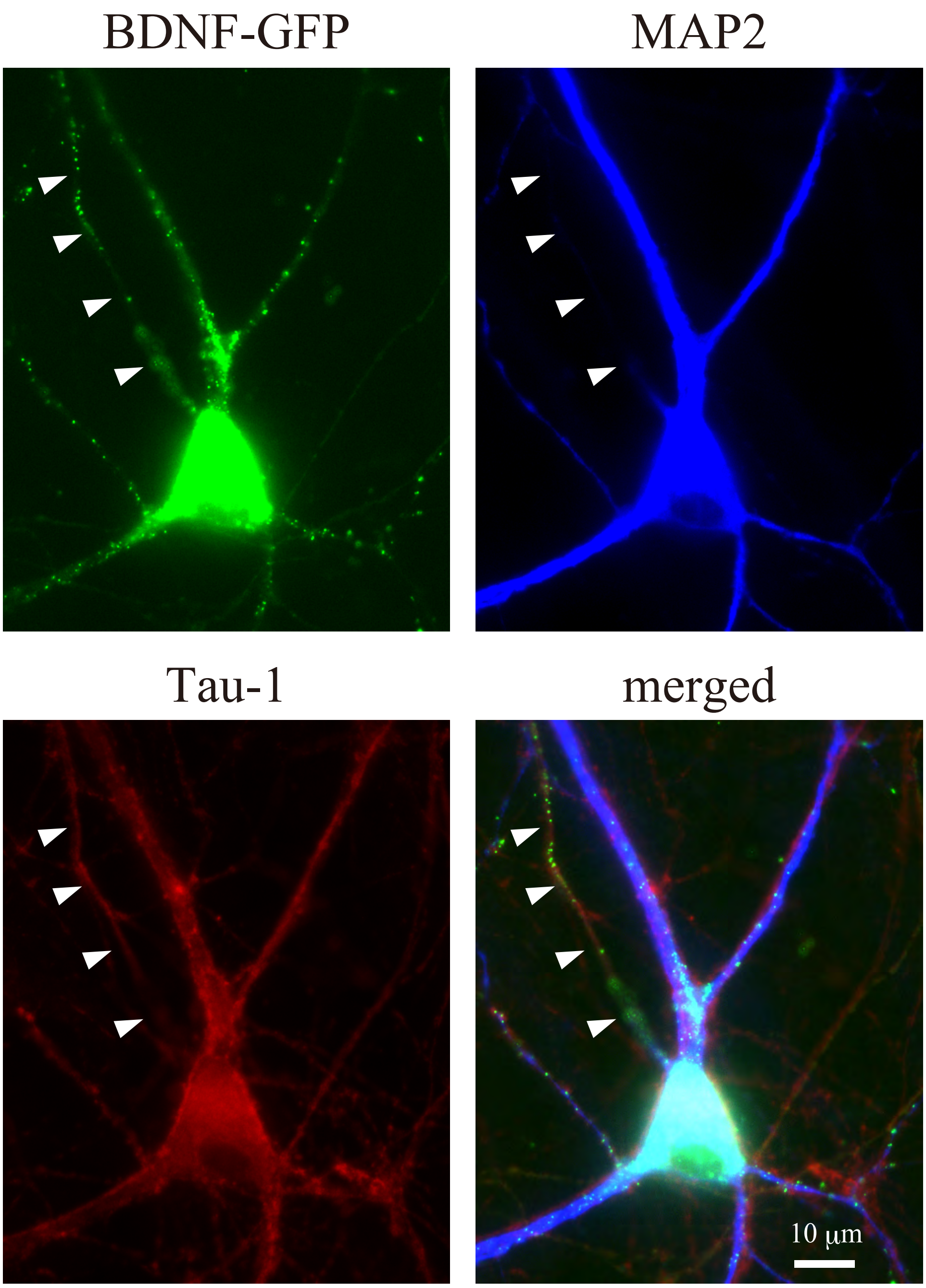

Title: Glucocorticoid affects dendritic transport of BDNF-containing vesicles

Naoki Adachi, Tadahiro Numakawa, Shingo Nakajima, Masashi Fukuoka, Haruki Odaka,
Yusuke Katanuma, Yoshiko Ooshima, Hirohiko Hohjoh, and Hiroshi Kunugi

Supplementary Figure S2

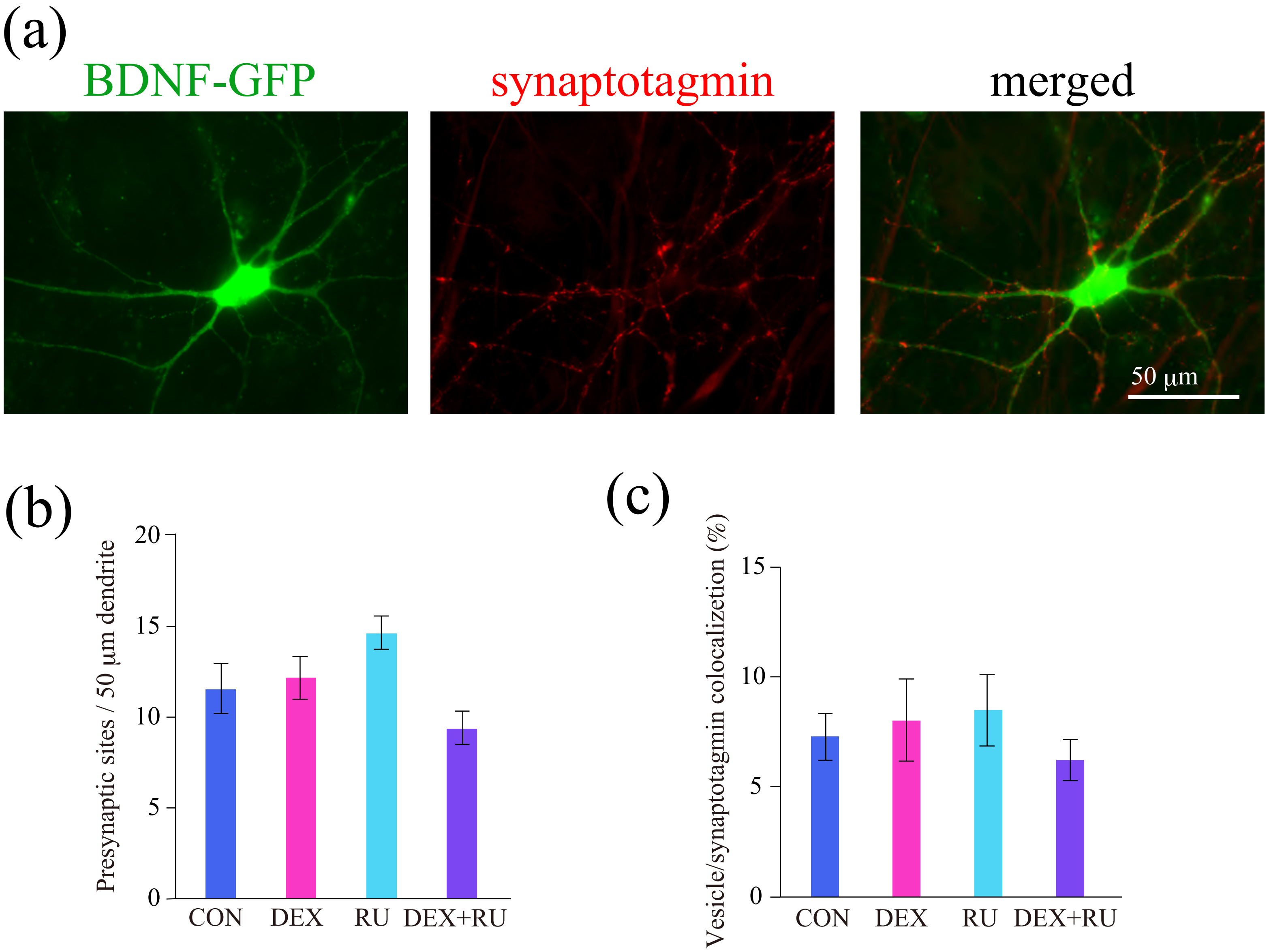

Title: Glucocorticoid affects dendritic transport of BDNF-containing vesicles

Naoki Adachi, Tadahiro Numakawa, Shingo Nakajima, Masashi Fukuoka, Haruki Odaka, Yusuke Katanuma, Yoshiko Ooshima, Hirohiko Hohjoh, and Hiroshi Kunugi

Supplementary Figure S3

fig. 4a huntingtin

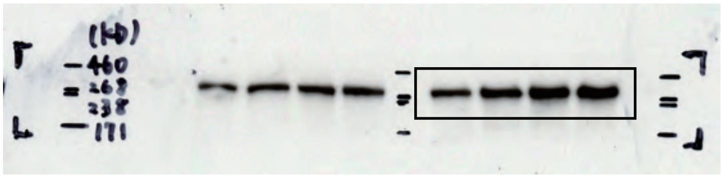

fig. 4a βActin

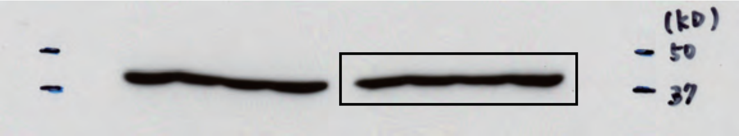

fig. 4b huntingtin

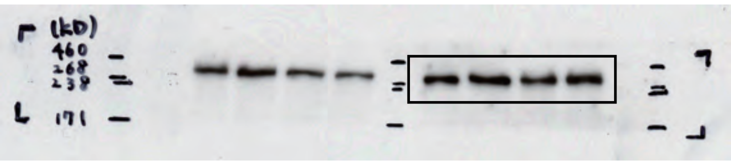

fig. 4b βActin

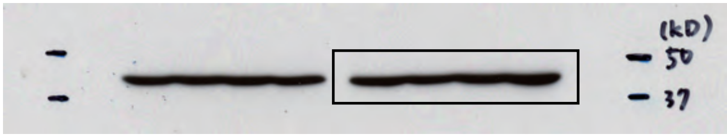

fig. 5c huntingtin

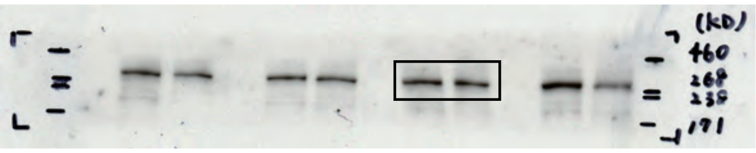

fig. 5c GFP

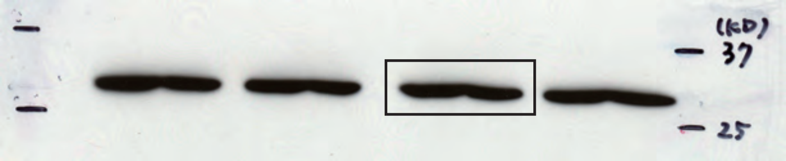

fig. 5c βActin

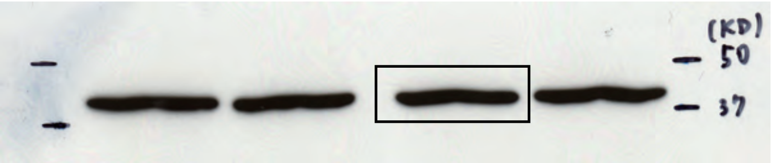

Supplement: Supplementary Information [file srep12684-s1.pdf]
